# Implementing a student-centered stroke intervention and prevention education program; evaluating motivation, cognitive load, and performance among middle school students

**DOI:** 10.3389/fpubh.2024.1332884

**Published:** 2024-04-16

**Authors:** Samuel Imeh-Nathaniel, Irraj Iftikhar, Ashley Snell, Katherine Brown, Keiko Cooley, Asa Black, Mohammed K. Khalil, Thomas Nathaniel

**Affiliations:** ^1^Department of Biology, North Greenville University, Tigerville, SC, United States; ^2^School of Medicine-Greenville, Greenville, SC, United States

**Keywords:** stroke, education, middle school, performance, cognitive load, motivation or difficulty

## Abstract

**Background:**

In this study, we investigated the association between motivation, cognitive load, difficulty, and performance in a stroke education outreach program implemented for middle school students.

**Methods:**

Various interactive instructional activities were developed to engage students throughout the program to assess cognitive and intrinsic load arising from learner implementation of various tasks in a stroke education program for middle school kids. Performance was measured using a post-test to assess knowledge gained by the 6th, 7th, and 8th-grade middle school students. A short questionnaire was also administered to collect data on students’ motivation using the ARCS model to asses attention, relevance, confidence, and satisfaction. In addition, we evaluated difficulty level and cognitive load. The relationship between performance and motivation was assessed using Pearson’s correlation.

**Results:**

In our results, there was no significant difference (*p* > 0.05) in performance between the 6th, 7th, and 8th-grade students. The difference in performance, cognitive load (mental effort and difficulty), or motivation between the 6th, 7th, and 8 t-grade students was not significant (*p* > 0.05). The correlation between motivation and performance was significant (*r* = 0.87, *p* = 0.001), while the correlation between mental effort and performance was not significant (*r* = 0.34, *p* = 0.270). Also, the correlation between difficulty and performance was not significant (*r* = 0.38, *p* = 0.361). In the ARCS motivation model, attention, and confidence received the lowest mean scores (3.9), while relevance received the highest score (4.3).

**Conclusion:**

Our findings reveal the importance of implementing novel activities to enhance students’ motivation to improve performance in the implementation of stroke education outreach programs for middle school students.

## 1 Introduction

Within the stroke belt, chronic diseases, poor diet, exercise, and lack of education on stroke awareness lead to an increasing stroke incidence (1). The Stroke Belt are regions in the Southeastern United States with higher stroke deaths from all strokes compared with other regions (2). The age-adjusted stroke mortality rate in the Stroke Belt is higher than for the other regions of the United States (3). Educating the younger generation is a way to avoid this cycle of lifestyle choices and will likely lead to fewer strokes in the future (4). It is only through proactive habits, such as healthy diets and exercise or physical activities, that children can learn how to avoid fatal strokes later in life (5). The use of exercise in the primary and secondary prevention of stroke has a growing evidence base and support. The maintenance of healthy habits and prevention of stroke has been linked with regular physical activity and balanced dietary intake (6). Among kids in middle school, sedentary habits such as watching television, lack of sports participation, and computer games are associated with a decline in physical activity throughout childhood (7). Therefore, given the significance of the decline in physical activity among middle school students, several school-based stroke education and physical health intervention programs (8, 9) have adopted different educational health promotion strategies among middle school students. In most of the existing studies (9, 10), how the employed instructional design, motivation, cognitive load, and difficulty, interplay with each other to impact learning and task performance in a stroke education outreach program implemented for middle school students is not known. Educational intervention must be tailored not just to suit the expertise levels, but also to meet learners’ motivational needs for the optimization of cognitive load and mental effort.

Most stroke education programs are within the context of cognitive load theory but have not considered the effect of learner affect and motivation on the use of cognitive capacity. Cognitive load level is typically determined by measuring perceived task difficulty (11), and this can also be the measure of learner motivation for the investment of cognitive effort in learning the materials in a stroke education program. Moreover, the perceived task difficulty can be associated with the perceived pleasure of learning the different tasks in the stroke education program (12). For example, middle school kids may perceive a learning task to be unpleasant if they are very difficult to learn, indicating high cognitive load during task performance, but pleasant and easier to learn, with a low cognitive load at the end. In other words, the structure of the learning tasks or activities in the stroke education program may alter the perception of a learning activity, to be high or low cognitive load, according to how middle school students feel about a particular learning activity. Therefore, the effect of perceived pleasure of a learning task needs to be considered in a stroke education program. Complex instructional techniques that interfere with learning may affect performance among early learners such as middle school students (13). A potential solution is to design learning materials that are less tasking, pleasant, and easier to learn especially for middle school learners in a stroke education program.

Cognitive load is about learning skills about solving problems that originate from extraneous cognitive load (instruction-related) (14). Extraneous cognitive load is described as reducing intrinsic cognitive load through instructional techniques that structure information to focus cognition on schema acquisition (15, 16). Cognitive load theory deals with solving academic tasks using innovative pedagogies (16, 17). Therefore, the cognitive load has dimensions that reflect the interface between learners, pedagogies, and evaluation that describes the measurable aspects such as mental load (ML), mental effort (ME), and performance (18). ML is task-related and represents the cognitive ability to process complex tasks (19). In comparison, ME measures the cognitive capability of an individual while the learner is implementing a specific task (19). Sweller et al. (16) suggested that ML and ME are interlinked. De Jong (14) proposed that performance is a component of cognitive load (CL) and sometimes serves as an indicator for CL. Measuring CL is an important factor to measure in an educational program (20–22). For example, CL can be assessed as a control factor to better understand students’ test performance in an educational program (23, 24). Therefore, the interaction between cognitive load and motivation as it relates to learning different activities in a stroke education program will depend on whether the different tasks are easy and pleasurable enough to motivate middle students for better task performance in a stroke education program.

Educational instruction, even when designed and based on effective instructional strategies may not necessarily stimulate students’ motivation to learn (25). This is because it is possible that students may not be motivated to pursue lifelong learning and use the knowledge and skills learned to implement measures related to the learned materials. The ARCS model is an instructional design model developed by John Keller (26) and focuses on motivation. The four elements; Attention, Relevance, Confidence, and Satisfaction form the acronym ARCS of the model. Attention is the extent to which the student’s curiosity is aroused or gained and sustained over time. Relevance indicates the student’s perception that the instruction is related to personal needs. Confidence is the student’s perceived likelihood of achieving success through personal efforts and control, and Satisfaction implies reinforcing accomplishment with rewards from the instruction, which can include internal and external factors. The ARCS model is well-validated and has been used in several education studies (27, 28) to help identify components of instruction that can increase student motivation to learn with responsive to the interests and needs of students. Using motivational design to create educational strategies and then incorporating these into the instruction materials can result in complementary enhancement of student learning and performance (29) in a stroke education program. In this study, we investigated the association between motivation, cognitive load, difficulty, and performance in a stroke education outreach program implemented for middle school students. This allows us to determine whether the available pleasurable amount of motivational resources in a stroke education program for middle school can promote better learning, cognitive load, and performance for middle school students. Our prospective findings would help better explain the effect of tailoring an instructional design with a pleasurable amount of resources in a stroke education program on the motivation and performance of middle school students in a stroke education program.

### 1.1 Study rationale

Understanding the relationship between cognitive load and motivation is important in educational learning activities (20, 30–32). This is significant especially since components of the instructional materials can actively manipulate students’ cognitive load or motivation (14). An important theory of motivation is the self-determination theory, which states that instructors should always seek to intrinsically motivate students, as it helps improve their competence (33, 34). Therefore, investigating motivation, cognitive load, and motivation in a stroke education program for middle school kids is important to determine their relationship and the impact on middle school kids during the implementation of interactive active learning activities in a stroke education outreach program. While active learning activities provide measures of cognitive load and motivation associated with small group interactive learning activities (35), it is not clear whether active learning during interactive social activities that facilitate a better understanding of stroke signs and symptoms can differentially improve performance, cognitive load, motivation, or difficulty among 6th, 7th, and 8th-grade students with different age categories who participated in a stroke education outreach program. The following research questions guided our study:

Research question 1: Is there a statistically significant difference in the performance, mental effort, difficulty, or motivation of 6th, 7th, and 8th-grade students?

*Hypothesis*: There is no significant difference between 6th, 7th, and 8th-grade students indicating that active learning during interactive social activities may not differentially improve performance, mental effort, difficulty, or motivation levels among 6th, 7th, and 8th -grade students with different age categories who participated in the stroke education outreach program.

Research Question 2: Is there a significant correlation between motivation, mental effort, difficulty and performance?

*Hypothesis*: There is a statistically significant correlation between motivation and performance indicating the association of active learning activities with performance or motivation among 6th, 7th, and 8th-grade students who participated in a stroke education program.

In this study, we determined whether, (1) motivation, mental effort, and difficulty differ between 6th, 7th, and 8th-grade students, (2) motivation, mental effort, and difficulty correlate with students’ performance, and (3) ARCS (attention, relevance, confidence, and satisfaction) model of motivation provide insight into improving stroke education for future generations. The present study contributes to the existing literature on how motivation, cognitive load (mental effort and difficulty), and academic performance differ among 6th, 7th, and 8th-grade students in a stroke education program.

## 2 Methods

### 2.1 Participants

A total number of 355 middle school students from 6th grade = 145, 7th grade = 137, 8th grade = 73 from two different middle schools participated in the study. Participants were diverse, from various socioeconomic statuses and races, and their ages range from 11 to 14 years. Two hundred and fifty-one middle school students were involved in the program for the first year, while 104 middle students participated in the second year of the program. The program ran for the full duration of the school day, from 8:00 AM–2:30 PM, and consisted of 6 periods that lasted 50 min each in both years of implementing the program. The approval for this study was obtained from the PRISMA Health institutional committee for ethics (approval number: Pro00074119).

### 2.2 Stroke education activities

The Stroke Education Day program consists of various learning activities that were implemented by medical students for middle school students in their gym at two different schools. The program started by showing videos, which shares the reflections by stroke survivors. A total of five learning stations were implemented for Stroke Education Day, with 10–15 students per station. Students rotated through each learning station at a time. At these stations, students participated in different interactive activities that were guided by at least three medical students who were present in each station to facilitate the learning process.

The first station was the FAST station with the goal to introduce middle school kids to the FAST signs of stroke (e.g., F=Face drooping, A = Arm weakness, S=Speech difficulty, T = Time to call 911). Middle school students learned the signs and symptoms of stroke with a large visual representation of each letter and activities corresponding to each letter. These activities included attempting to mimic facial droop, holding a weight in one arm to simulate arm weakness, saying a tongue twister to simulate speech difficulty, and imitating calling 911 to emphasize how crucial time is when someone is having a stroke. By associating each letter of FAST with a stroke symptom, middle school students learned the signs and symptoms of stroke interactively.

The second station was “ReThink Your Drink,” in which middle school students were educated about added sugars and taught that limiting sugar intake could help prevent stroke. Specifically, the students were taught the amounts of sugar that can be found in everyday drinks. To facilitate an interactive environment to maximize student engagement, this station included an activity where students were asked to guess the amount of sugar in each drink and match the corresponding drink with bags filled with sugar.

Station three, named “The Salty 6,” aimed to educate middle school students about the salt content in different foods and how maintaining a healthy diet may help prevent stroke. At this station, middle school students learned the implication of high-level sodium, which is a risk factor for stroke. Middle school students were then able to participate in an activity where they learned the six saltiest foods in the American Diet (e.g., breads and rolls, pizza, sandwiches, cold cuts and cured meats, soup, burritos and tacos) and were able to try guessing how much sodium was in each of these foods.

The fourth station was an activity station, in which students could participate in various exercise activities, including racing, jumping, and pushups. The goal of this station was to emphasize the importance of exercise and physical activity in living a healthy lifestyle and preventing stroke. It was also a way to show students that there are a variety of ways that they can stay active and reach the AHA-recommended levels of weekly physical activity.

In the last station, “Life’s Simple 7,” middle students participated in a discussion with their peers and the medical students. At this station, students learned the seven habits of a healthy and unhealthy lifestyle, which include stop smoking, healthy diet, physical activity, lose weight, manage blood pressure, control cholesterol, and reduce glucose.

### 2.3 Data collection and analysis

**Figure 1:**
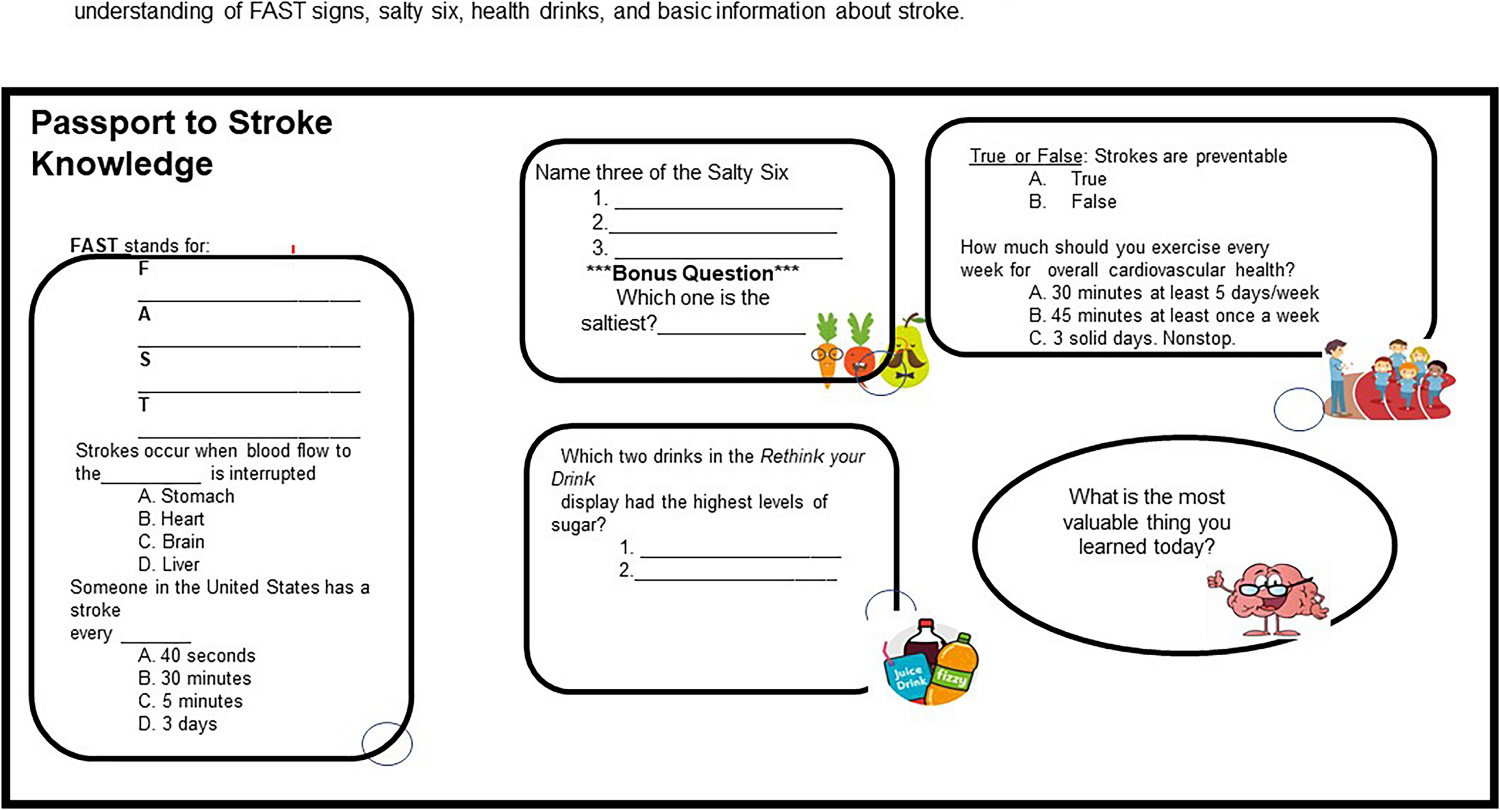
Individual students’ performances were assessed based on their responses to the questions that tested their understanding of FAST signs, salty six, health drinks, and basic information about stroke.

To evaluate the impact of the stroke program on middle school students to learn about measures to prevent stroke, we administered a survey at the completion of the learning activities (Table 1). The survey has been used in previous studies (6, 36) and was modified to reflect data collection for middle school students for our stroke education program. The first 8 items were based on the ARCS model of motivation (26). There are four components of the ARCS model that assesses attention, relevance, confidence, and satisfaction. Questions 9 and 10 correspond to mental effort and difficulty level as a measure of cognitive load. It is important to point out that mental effort and task difficulty are both subjective measures of cognitive load (37). However, they measure different aspects of cognitive load. The task difficulty rating is related to intrinsic load, and the mental effort is related to germane load (38). Individual students’ performance was assessed based on their responses to the questions in the Passport to Stroke (Figure 1). This allowed them to identify and recognize the FAST signs, salty foods, healthy drinks, and basic information about stroke and healthy lifestyles. After the data was collected, Cronbach’s alpha score was calculated and found to be 0.96 reflecting that the survey was internally consistent. Next, we determined the percentages of students in the 6th, 7th, and 8th-grades and their responses to the questionnaire. In addition, the means, and standard deviations (SD) for all variables were calculated. To determine the difference in performance, mental effort, motivation, or difficulty between the 6th, 7th, and 8th-grade students, we used a one-way ANOVA. In using ANOVA, we considered the independence of the groups being compared. This allowed us to determine whether there is a statistically significant difference in the performance, mental effort, motivation, or difficulty between 6th, 7th, and 8th-grade students. We tested for the normal distribution of our data using exploratory data analysis (EDA) before the use of the parametric test. Finally, the relationship between performance, mental effort, motivation, and difficulty was determined using Pearson’s correlation coefficient. The significance level was established at *p* < 0.05. We analyzed all our data using SPSS version 29 (SPSS; IBM Corporation, Armonk, NY, United States).

**Table 1:** Measures of motivation using ARCS model (attention, Q 1–2; relevance, Q 3–4; confidence, Q 5–6; and satisfaction, Q 7–8) and cognitive load including mental effort and difficulty in middle school students who participated in the stroke education program.

Stroke Education Activities (SEA)					
	Very true				Not true
1. The SEA session kept me excited to complete the assigned activities	5	4	3	2	1
2. The SEA experience aroused my curiosity	5	4	3	2	1
3. The content I am learning during SEA session will be useful to me	5	4	3	2	1
4. Students actively participated in the SEA assigned activities	5	4	3	2	1
5. The information to be learned during SEA is just too difficult for me	5	4	3	2	1
6. I found the challenge of learning about stroke by SEA to be about right	5	4	3	2	1
7. Completing SEA tasks gave me a satisfying feeling of accomplishment	5	4	3	2	1
8. I enjoyed the SEA experience	5	4	3	2	1
9. Indicate the amount of mental effort you invested during the stroke learning activities					
Very low mental effort					
Low mental effort					
Rather low mental effort					
Neither low nor high mental effort					
Rather high mental effort					
High mental effort					
Very high mental effort					
10. Indicate how easy or difficult the stroke teaching activities were					
Extremely easy					
Easy					
Somewhat easy					
Neither easy nor difficult					

5 = very true, 4 = mostly true, 3 = moderately true, 2 = slightly true, and 1 = not true.

## 3 Results

For two years, 355 middle school students participated in the stroke education outreach program. Of the 355 students, 145 (40.8%) were in the 6th grade, 137 (38.6%) were in the 7th grade, and 73 (20.6%) were in the 8th grade. Table 2 presents the results for performance, mental effort, motivation, and difficulty. The average score for students in the 6th grade was 76.3%, 7th grade was 73.6%, and 8th grade was 68.7%. The difference in performance between the 6th, 7th, and 8th-grade students was not significant (*p* > 0.05). Moreover, the difference between the different grade levels in mental effort, motivation, or difficulty was also not significant (*p* > 0.05). Although the correlation between motivation and performance was significant (*r* = 0.87, *p* = 0.001), neither the correlation between mental effort and performance was not significant (*r* = 0.34, *p* = 0.270) nor between difficulty and performance (*r* = 0.38, *p* = 0.361).

**Table 2:** Measurements of mental effort, difficulty, motivation, and performance of middle school students who participated in the stroke education program between 2018 and 2019.

	6th grade (*N* = 145)	7th grade (*N* = 137)	8th grade (*N* = 73)	*p* value
	Mean (SD)	Mean (SD)	Mean (SD)	
Mental effort	4.57 (1.49)	4.57 (1.78)	4.10 (1.56)	0.106
Difficulty	1.96 (1.04)	1.97 (1.18)	2.15 (1.31)	0.491
Motivation	30.9 (7.52)	31.6 (7.20)	31.0 (5.64)	0.722
Performance	76.3 (25.0)	73.6 (29.2)	68.7 (25.8)	0.163

Table 3 presents the results of the ARCS model of motivation. The average result for attention (“A”—questions 1&2) was 3.9, relevancy (“R”—questions 3&4) was 4.3, confidence (“C”—questions 5&6) was 3.9, and satisfaction (“S”—questions 7&8) was 4.1. The ratings for all four categories of the ARCS model were above 4/5 indicating a high level of motivation during the learning activities. Relevance received the highest rating. 84.4% of all the students noted that it was either mostly or very true that the content they learned during the stroke education activities (SEA) would be useful to them.

**Table 3:** The motivation scores based on the ARCS model for middle school students who participated in the stroke education program between 2018 and 2019.

Survey Items	Not true/slightly true *N* (%)	Moderately true *N* (%)	Mostly true/very true *N* (%)	Mean (SD)
1. The SEA session kept me excited to complete the assigned activities	27 (8.5)	82 (25.9)	207 (65.5)	3.9 (1.1)
2. The SEA experience aroused my curiosity	33 (10.5)	78 (24.8)	203 (64.6)	3.9 (1.2)
3. The content I am learning during SEA session will be useful to me	17 (5.5)	31 (10.1)	260 (84.4)	4.4 (0.9)
4. Students actively participated in the SEA assigned activities	21 (6.9)	45 (14.9)	237 (78.2)	4.2 (1.0)
5. The information to be learned during SEA was not too difficult for me	73 (24.7)	27 (9.2)	195 (66.1)	3.8 (1.6)
6. I found the challenge of learning about stroke by SEA to be about right	43 (14.4)	50 (16.8)	205 (68.8)	3.9 (1.3)
7. Completing SEA tasks gave me a satisfying feeling of accomplishment	38 (12.6)	55 (18.3)	208 (69.1)	4.0 (1.2)
8. I enjoyed the SEA experience	31 (10.1)	48 (15.6)	228 (74.3)	4.1 (1.2)

## 4 Discussion

This study investigated whether motivation, mental effort, difficulty, and performance differ among 6th, 7th, and 8th-grade students and how this provides insight into improving stroke education among middle school students. Three major findings originated from our study. First, there was no significant difference in performance, cognitive load (e.g., mental effort and difficulty), or motivation between the 6th, 7th, and 8th-grade middle school students. Second, we observed a significant correlation between motivation and performance, but we did not observe a significant correlation between cognitive load and performance. Finally, students’ motivation was high at the end of the stroke education outreach program using the ARCS model, and relevance received the highest rating.

In our study, students’ motivation was high at the end of the stroke education outreach program using the ARCS model, and relevance received the highest rating, indicating students’ perception that the instruction is related to their personal needs in learning different measures to prevent stroke. It is possible to change the learning conditions and environment so that each student can motivate themselves to learn the activities in the stroke education program. Even in the changed environment, it is also possible that the medical students and facilitators of the program can teach, and teach well, without having middle school students learn the materials. In addition, middle school students may reluctantly learn things that do not mean anything to them. They also may choose not to use or apply what they have learned in the stroke education program to improve healthy lifestyles. The motivational strategies used in this study allowed us to positively influence middle school students’ motivation to learn materials that can help prevent strokes. Our finding of increased motivation has been reported by other studies that support the validity of the four basic constructs and their positive effects on student motivation and performance (29, 36). In addition, our finding reveals the importance of using the ARCS Model to evaluate the effectiveness of educational strategies in a stroke outreach program for middle school students.

In our program, medical students as facilitators implemented different healthy lifestyle interactive activities. They led the conversations in their small groups and shared their ideas and thoughts on different activities they embarked upon at home including exploring their local parks, biking, hiking, walking on street trails, outdoor tennis or volleyball courts, or baseball and football fields. They shared experiences of how their daily routines help develop life skills, reduce stress, and build healthy habits. They provided specific examples such as assisting with laundry and washing dishes. They worked together, interacted socially, and had fun while implementing the different activities that kept them physically active in the stroke education outreach program. They were motivated to implement physical activities that allow them to live healthy lifestyles and help prevent stroke in the future. In general, when middle school kids are provided with the opportunity to take responsibility for their learning and provided with interactive activities they could perceive the entire learning activities as fun instead of mainly educational.

A major finding of this study is that performance, cognitive load, or motivation between the 6th, 7th, and 8th-grade students were not significantly different. This finding suggests that our learning strategy may provide some innovations in motivating middle school students of a broad age category to be actively involved in their learning by working together during the different stroke learning activities, identifying problems, and paired discussions, with a greater responsibility in solving the problem on their own. This approach allows higher-order thinking, with emphasis on student’s ability to solve problems, struggle with complicated issues such as signs and symptoms of stroke, suggest solutions on how to prevent stroke and explain ideas in their own words to their colleagues. Therefore, our approach of using interactive visual learning materials not only improves learning in middle school students across 6th 7th, and 8th -grade classes but also cognitive load level was appropriate for all the three student grades who participated in the program (39).

There was a significant correlation between motivation and performance, and motivation was high at the end of the stroke education program. For academic success, motivation is known to be an important variable (40). This is because it enhances the willingness of the learner to implement a specific task (41). Motivation can be intrinsic and extrinsic (42). The latter form of motivation focuses on the benefit of a specific reward while the learner is implementing a task (43). An example of extrinsic motivation is when a student is motivated by reviewing materials primarily to perform well in an examination (44). Intrinsic motivation is important in the academic environment (42, 45, 46). An example of intrinsic motivation implemented in this program is students learning healthy lifestyles and stroke prevention activities because the topic is fun and the learning materials are entertaining. This type of intrinsic motivation is proposed to increase performance among learners (47). This finding supports our current result and indicates that intrinsic motivation may be associated with using an excellent instructional strategy for middle schoolers that is entertaining, with unforgettable experiences to help develop healthy lifestyles.

While students’ motivation was high at the end of the stroke education program, we observed that relevance received the highest rating. The high rating of relevance in our ARCS model of motivation suggests students’ awareness that participation in this program can equip them with the knowledge to save the lives of their friends and family in the future. Therefore, future stroke education outreach programs may consider strategies to increase students’ confidence and attention. For example, improving the student’s confidence because students with higher confidence levels tend to be more attentive and involved (26). This indicates that attention can be dependent on confidence. Keller suggests various strategies for working on students’ confidence, including organizing activities on an increasing difficulty level and linking students’ successful performance to their hard work (26).

Furthermore, developing activities that stimulate and sustain the students’ attention, rather than simply capturing it initially, may be an effective strategy (48). Some specific examples include presenting the students with various problem-solving situations to increase participation, shifting between instructor-student interaction and student–student interaction to increase variability, and using humorous analogies.

### 4.1 Limitations

There are some limitations in this study. For example, the lack of control groups and data were collected from only two middle schools. There was no pre-test given to the students; therefore, there is no way to see if there was a major gain in learning the stroke signs, symptoms, risk factors, and prevention after participating in the stroke education program. Another limitation is that several students did not answer all of the questions on the survey. This may be due to their young age, lack of maturity, or possibly even a lack of time to complete the survey. In addition, single-item assessments used in this study that include student self-assessments are generally less reliable and statistically sound especially in measuring cognitive load and difficulty that likely would have been more appropriate. No test or survey was established to give to students who previously participated in the program. We did not code the different comments made by students during the sessions, to generate qualitative data for the sessions. However, longitudinal data could provide insight into whether students retain the knowledge gained during the program and whether it has proven helpful in their lives. In this study, we used Cronbach’s alpha to measure the reliability but not validity. While it helped us to determine whether responses are consistent between items (reliability), it did not determine whether the items measure the correct concept (validity).

## 5 Conclusion

The current study developed stroke education program that adopt interactive learning strategies that allowed middle school students to initially watch videos made by stroke survivors and then participate in small group interactive learning activities. The interactive social activities were designed to enable active learning activities in the learning of stroke signs and symptoms, prevention, and promoting healthy lifestyle activities. In addition, active learning activities provided measures that reflect changes in cognitive load and motivation originating from the small group interactive learning activities. Our findings indicate that the different activities implemented in the stroke program are sensitive to changes in motivation and optimize the difficulty level to avoid cognitive overload among middle schoolers engaged in the stroke education program.

## Data availability statement

The original contributions presented in the study are included in the article/supplementary material, further inquiries can be directed to the corresponding author.

## Ethics statement

This study was approved by the Institutional Committee for Ethics (approval number: Pro00074119). The studies were conducted in accordance with the local legislation and institutional requirements. Written informed consent for participation in this study was provided by the participants’ legal guardians/next of kin.

## Author contributions

SI-N: Conceptualization, Formal analysis, Investigation, Methodology, Writing – original draft. II: Investigation, Methodology, Writing – review & editing. AS: Investigation, Methodology, Writing – review & editing. KB: Investigation, Methodology, Writing – review & editing. KC: Investigation, Methodology, Writing – review & editing. AB: Methodology, Investigation, Writing – review & editing. MK: Conceptualization, Formal analysis, Investigation, Methodology, Writing – original draft. TN: Formal analysis, Funding acquisition, Investigation, Methodology, Supervision, Writing – original draft, Writing – review & editing.
